# Identification of novel small molecule Beclin 1 mimetics activating autophagy

**DOI:** 10.18632/oncotarget.17977

**Published:** 2017-05-18

**Authors:** Jia Yu, Lan Lan, Seth J. Lewin, Steven A. Rogers, Anuradha Roy, Xiaoqing Wu, Philip Gao, John Karanicolas, Jeffrey Aubé, Baiwang Sun, Liang Xu

**Affiliations:** ^1^ School of Chemistry and Chemical Engineering, Southeast University, Nanjing 210089, China; ^2^ Department of Molecular Biosciences, The University of Kansas, Lawrence, Kansas 66045, USA; ^3^ Center of Biomedical Research Excellence, The University of Kansas, Lawrence, Kansas 66045, USA; ^4^ High Throughput Screening Laboratory, The University of Kansas, Lawrence, Kansas 66045, USA; ^5^ COBRE-PSF Protein Production Group, The University of Kansas, Lawrence, Kansas 66045, USA; ^6^ Center for Bioinformatics, The University of Kansas, Lawrence, Kansas 66045, USA; ^7^ Department of Medicinal Chemistry, The University of Kansas, Lawrence, Kansas 66045, USA

**Keywords:** Beclin 1 mimetics, autophagy, Bcl-xL protein, cancer, high-throughput screen

## Abstract

Anti-apoptotic proteins Bcl-2 and Bcl-xL could block autophagy by binding to Beclin 1 protein, an essential inducer of autophagy. Compounds mimicking Beclin 1 might be able to disrupt Bcl-xL/2-Beclin 1 interaction, free out Beclin 1, and thus trigger autophagy. In order to identify small molecule Beclin 1 mimetics, a fluorescence polarization-based high-throughput screening of 50,316 compounds was carried out with a Z’ score of 0.82 ± 0.05, and an outcome of 58 hits. After the structure analysis, three acridine analogues were unveiled and confirmed using the fluorescence polarization assay and the surface plasmon resonance assay. Moreover, a set of 17 additional acridine analogues was prepared and tested. Compound 7 showed selectivity for Bcl-xL (*K_D_* = 6.5 μM) over Bcl-2 (*K_D_* = 160 μM) protein, and potent cytotoxicity (nanomolar scale) in PC-3, PC-3a and DU145 prostate cancer cells. Furthermore, induction of autophagy was also demonstrated in PC-3 and PC-3a cells treated with some acridine compounds by LC3 conversion immunoblotting and LC3 fluorescence microscopy. These Beclin 1 mimetics will be invaluable tools for developing novel autophagy inducers, better understanding the roles of autophagy in cancer, and will contribute to cancer therapy.

## INTRODUCTION

Autophagy is an essential, evolutionarily conserved process that degrades protein aggregates and damaged organelles in order to maintain cytoplasmic homeostasis [1]. It begins with the sequestration of cytoplasmic constituents in double-membrane vesicles, called autophagosomes. The vesicles then fuse with lysosomes to form autolysosomes, where the engulfed contents undergo degradation and recycling to sustain cellular metabolism [2].

In normal cells, autophagy is generally considered as a cytoprotective mechanism [1]. However, the roles of autophagy in cancer cells are paradoxical and controversial. Autophagy acts both as a tumor suppressor that prevents tumor initiation and as a protector of cancer cell survival that removes damaged organelles under stress conditions [3, 4]. The outcome of autophagy regulation in cancer cells may vary depending on the types of cancers, individual characteristics of cancer cells, microenvironments, and therapeutic treatments [5, 6]. Under certain circumstances, overactivation of autophagy can lead to autophagic cell death, or type II programmed cell death [6, 7]. In addition, most of the cellular systems where autophagy has been proven to contribute to cell death are defect in the apoptosis signaling pathways, as is the case in most tumors [8, 9]. Therefore, even though autophagy is most likely a pro-survival response in normal cell homeostasis, it might be a backup death program in apoptosis-resistant tumor cells [7, 9, 10]. If this hypothesis is correct, drugs that specifically trigger autophagic cell death should be well-tolerated due to highly specific killing of cancer cells. Moreover, autophagic cell death is a tempting way to circumvent apoptosis-resistance [11–13].

Autophagy is regulated by a set of conserved proteins, the autophagy-related (Atg) proteins [14]. Beclin 1, also named Atg6, is the first identified mammalian Atg protein and an essential autophagy inducer [15]. Beclin 1 forms a regulatory complex with class III phosphatidylinositol-3-kinase (class III PI3K or VPS34) that is important in the initiation of autophagy [16]. Beclin 1 is also a haploinsufficient tumor suppressor and mono-allelic deleted in 40%–75% human breast, ovarian and prostate cancer [15, 17]. Bcl-2 (B cell lymphoma 2) family proteins have been proposed as dual regulators of apoptosis and autophagy [18]. The anti-apoptotic Bcl-2 homologs, Bcl-2 and Bcl-xL, can downregulate autophagy by interacting with the Beclin 1/class III PI3K complex, and sequestering Beclin 1 [16, 19, 20]. Beclin 1 carries one Bcl-2-homology-3 (BH3) domain, which is important for the Bcl-2-Beclin 1 interaction and required for Bcl-2-mediated inhibition of autophagy [21, 22].

Several Bcl-2 inhibitors have been proven to competitively disrupt the interaction between Beclin 1 and Bcl-2/xL, and induce autophagy. (–)-Gossypol is the first Bcl-2 inhibitor approved by FDA for clinical testing (AT-101) and has entered Phase II-IIb clinical trials (www.ClinicalTrials.gov). In our previous studies, we demonstrate that (–)-gossypol preferentially induces autophagy in androgen-independent or castration-resistant prostate cancer cells that have high levels of Bcl-2 and are resistant to apoptosis, whereas apoptosis is preferentially induced in androgen-dependent cells with low Bcl-2 [8]. (–)-Gossypol induces autophagy *via* blocking Bcl-2-Beclin 1 interaction at the endoplasmic reticulum [8]. Moreover, the Bcl-2 inhibitor ABT-737 induces high levels of autophagy *via* binding to the BH3 binding groove of Bcl-2 or Bcl-xL (but not Mcl-1) and freeing out Beclin 1 [23–25]. ABT-737-induced autophagy cannot be inhibited by Bcl-2 or Bcl-xL overexpression, yet it is abolished by transfection with Mcl-1 or by the siRNA mediated knockdown of Beclin 1 [23, 26]. ABT-737 and its analogue ABT-263 (navitoclax) are in phase I/ phase II clinical trials [27].

In our previous screen for inhibitors of Bcl-2, we have discovered and synthesized a series of small molecule BH3-mimetics, including (–)-gossypol [8, 28]. We hypothesize that, in our original screening, we only focused on inhibitors of Bcl-2-Bax/Bak interaction that induce apoptosis, thus we may have missed some potential hits that more potently inhibit Bcl-2/xL-Beclin 1 interaction and induce autophagy instead of apoptosis. Here we report the discovery of several acridine compounds as small molecule Beclin 1 mimetics using the fluorescence polarization-based (FP-based) high-throughput screening (HTS), the surface plasmon resonance (SPR) assay, and cell-based biological activity assays. The acridine compounds have inhibitory effects on the proliferation of prostate cancer cells and induce autophagy. These Beclin 1 mimetics represent promising leads to develop novel molecular therapy for human cancer with Bcl-2/xL overexpression and resistant to conventional chemo/radiotherapy.

## RESULTS

### FP-based HTS for Beclin 1 mimetics

**Figure 1:**
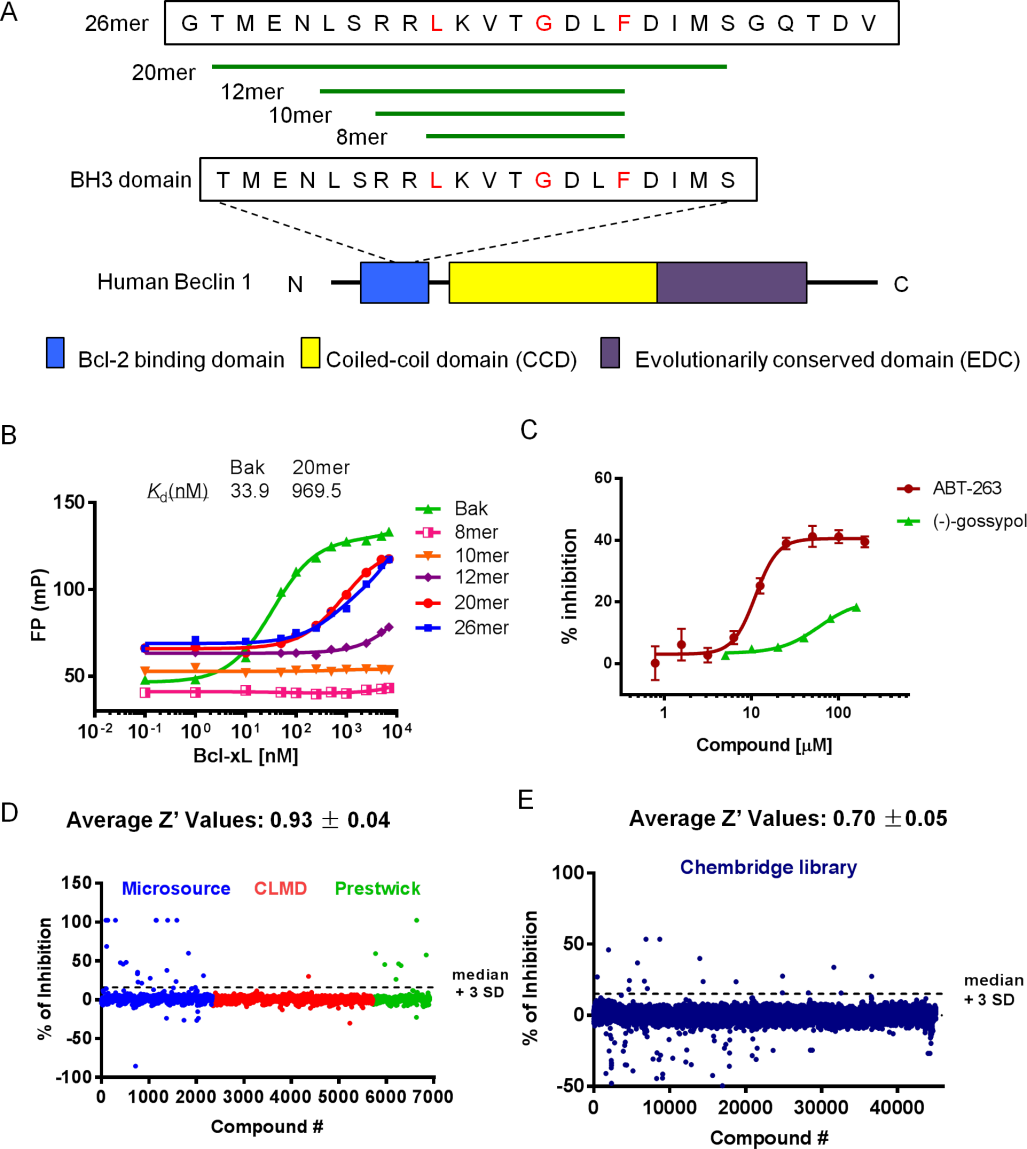
FP-based HTS for Beclin 1 mimetics (**A**) Schematic presentation of the Beclin 1 protein and sequences of five synthetic peptides modified from Beclin 1 BH3 peptide. Diagram of human Beclin 1 protein with different domains is shown in the bottom. BH3 domain is necessary and sufficient for interaction with Bcl-2/Bcl-xL. Amino acids in red are the critical residues for the binding. (**B**) Five fluorescein-labeled Beclin 1 BH3 peptides were tested for their binding ability to Bcl-xL protein in FP assay. Fluorescein-labeled Bak BH3 peptide was used as a positive control. Bcl-xL protein was titrated to a solution of 50 nM peptides. (**C**) Competitive binding assay with positive controls, ABT-263 and (–)-gossypol ((–)G). Compounds were titrated to a solution of preformed 20-mer/Bcl-xL complexes. The concentration was 50 nM for 20-mer and 2 μM for Bcl-xL protein. (**D–E**) HTS was performed with ~50000 compounds from Prestwick, Microsource, CMLD and Chembridge libraries. The four libraries were divided into two groups and screened separately. Compounds activity (percent of inhibition) of first screen (D) and second screen (E) were shown with Z′ value indicated on top. Plate median + 3SD was used as a threshold to determine the initial hits.

To discover small molecule inhibitors of Bcl-xL-Beclin 1 interaction, a sensitive, quantitative, *in vitro* FP-based binding assay was established and optimized for HTS. We first determined the proper Beclin 1 peptide for the FP assay. BH3 domain is necessary and sufficient for the interaction between Beclin 1 and Bcl-xL [22]. We therefore designed five fluorescein-labeled Beclin 1-BH3 peptides with different length. They all contain the critical residues for the binding behavior (Figure 1A). A 16-mer (a 16 amino acid long peptide) Bak-BH3 peptide was used as a positive control [29]. As shown in Figure 1B, Bcl-xL was titrated to a fixed solution of 50 nM fluorescent peptide and the changes of FP values were calculated. 20-mer Beclin peptide, 26-mer Beclin peptide as well as Bak peptide exhibited good binding curves to Bcl-xL in this assay, while peptides of shorter sequence (e.g. 8-mer, 10-mer and 12-mer) showed low or no binding affinity. Thus, we chose 20-mer, the shorter one of the two active peptides, as the binding peptide in the following FP assay. Buffer condition was then optimized (data not shown). Finally, 20-mer and Bak peptide was demonstrated to bind to Bcl-xL protein with equilibrium dissociation constants (*K*_d_) of 969.5 nM and 33.92 nM, respectively (Figure 1B), which are sufficient for the following FP-based assays. Then, we set up a FP-based competitive binding inhibition experiment using 50 nM of fluorescent 20-mer and 2 μM of Bcl-xL (Figure 1C). Positive compounds, ABT-263 [30, 31] and (–)-gossypol [12] are capable of freeing out fluorescent 20mer to experimental solution, and ABT-263 is more potent than (–)-gossypol (Figure 1C). These results demonstrated that this FP competitive binding assay is suitable for identify small molecule inhibitors that can mimic Beclin 1 and abrogate Bcl-xL-Beclin 1 interaction.

Next, we optimized the FP competitive binding assay in a 384-well microplate format and carried out a small-scale HTS using a small library, which contains 6580 compounds from Prestwick, Microsource and our in-house CMLD library. Then, the FP assay was optimized and applied in screening the larger Chembridge library consisting of 43736 compounds. The scattergrams of inhibitors activity are shown in Figure 1D–1E. HTS cascades are shown in Supplementary Figure 1A–1B. According to the percent of inhibition, 68 active compounds (> median + 3SD) were selected as initial hits. After removal of autofluorescent and repeated compounds, 41 hit compounds from the first screen (A01-A41) and 17 hit compounds from the second screen (B01-B17) were chose for further studying, giving an overall hit rate of 0.12% (Supplementary Table 1). The average Z′ value was 0.82 ± 0.05 with a signal to background window of 1.4, indicating a robust and reliable HTS assay (Figure 1D–1E; Supplementary Figure 1C–1F). A complete list of the screening hits and the confirmatory data were listed in Supplementary Table 2–3 with PubChem Chemical ID (CID) number. All chemical structures can be found on the PubChem website (http://pubchem.ncbi.nlm.nih.gov/).

### Structural similarity analysis of HTS hits

**Figure 2:**
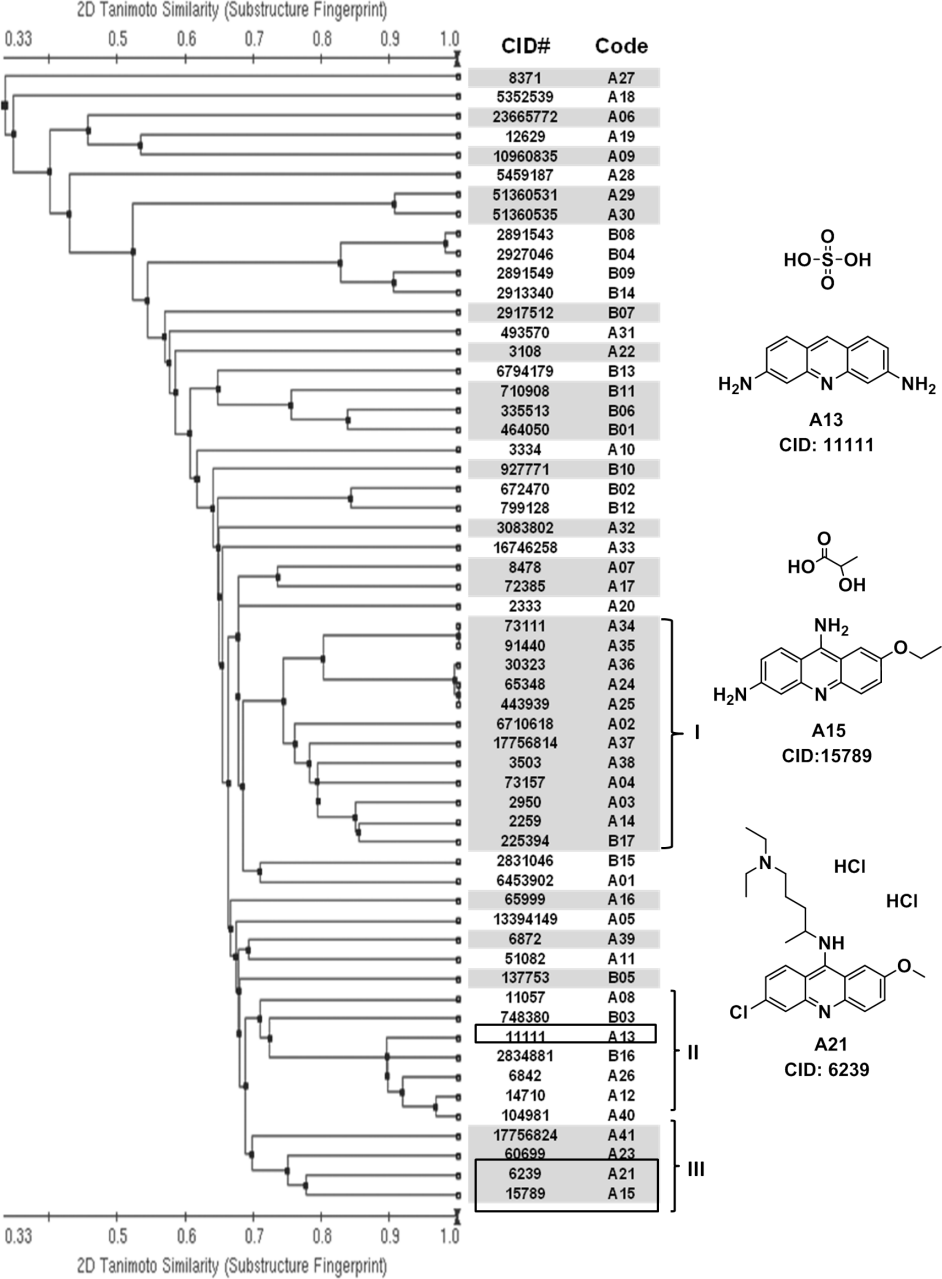
Structural similarity analysis of HTS hits Left panel: based on the structure (fingerprint) similarity, 58 hits were sorted using a free online clustering tool from PubChem (http://pubchem.ncbi.nlm.nih.gov/). Single-linkage algorithm was used in the clustering, and a Tanimoto distance of 0.3 resulted in 29 different clusters and singletons (shown in white and grey). Compounds are listed by their CID number and code. Chemical structures can be found on the PubChem website, using their CID. Group I, II and III are three major clusters. Right panel: chemical structures of promising candidate compounds from cluster II and III. They contain a common structure of acridine.

We employed a chemical structure clustering tool, a free online service from PubChem, to access the structure similarity of our hits. Tanimoto coefficient was used to quantify the structure similarity, which is by far the most popular and in widespread use today in computational medicinal chemistry [32]. Structural similarity clustering of 58 hits by a Tanimoto distance of 0.3 resulted in 29 different clusters and singletons. Three major clusters were revealed (Figure 2). We found that some compounds in cluster I might be promiscuous, assay-duping molecules. They have the common structure of polyhydroxy naphthalene or anthraquinone, therefore can be considered as pan-assay interference compounds, or PAINS [33]. After carefully looking over the hit structures, three acridine analogues, A13, A15 and A21, from cluster II and cluster III were subjected to further analysis (Figure 2).

### Validation of acridine compounds using two biochemical assays

**Figure 3:**
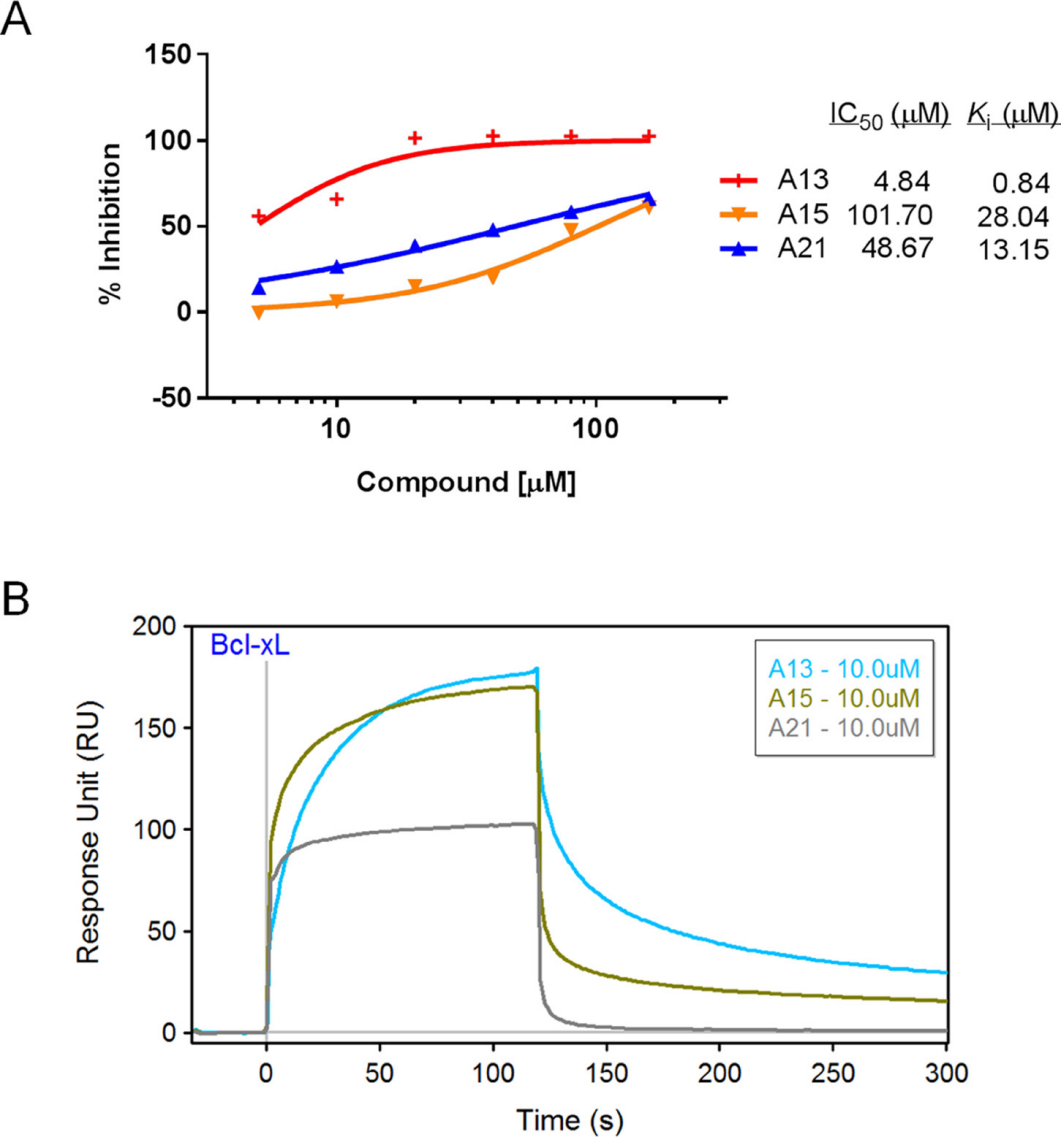
Validation of compounds A13, A15, and A21 binding to Bcl-xL protein (**A**) Dose-response curves of acridine compounds disrupting Bcl-xL-20-mer complex in FP assay using 2.5 μM Bcl-xL protein and 400 nM fluorescein-labeled Beclin 1 BH3 peptide (20-mer). (**B**) SPR sensorgram of acridine compounds confirmed their binding abilities to immobilized Bcl-xL protein at the concentration of 10 μM. Bcl-xL protein was immobilized on the sensor chip with 10000 RU.

We first validated the abilities of three acridine analogues to disrupt Bcl-xL-20-mer complex using the FP assay. As shown in Figure 3A, all three compounds exhibited potent and dose-dependent inhibitory effects in this assay (A13, *K*_i_ = 0.84 μM; A15, *K*_i_ = 28.04 μM; A21, *K*_i_ = 13.15 μM).

Next, we developed a surface plasmon resonance assay to validate the binding activities of these compounds. SPR is a label-free, non-fluorescent biochemical assay that could assess the binding behavior directly. Bcl-xL protein was immobilized to the sensor surface, and analytes in solution were injected over the surface. As shown in Figure 3B, compounds A13, A15, and A21 could bind to Bcl-xL at the concentration of 10 μM, and the SPR sensorgram displayed direct interactions between compounds and Bcl-xL protein. For example, while A21 was injected over the surface, response unit (RU) increased rapidly, indicating a fast accumulation or binding of A21 on the surface; while A21 was diluted away by running buffer, RU decreased rapidly, indicating a fast dissociation of the complex. Obviously, A21 dissociated faster than the other two acridine compounds, indicating the different interactions between Bcl-xL and the acridine compounds (Figure 3B). Taken together, FP and SPR results confirmed that compounds A13, A15, and A21 could abrogate Bcl-xL-Beclin 1 interaction through direct binding to Bcl-xL protein.

### Acridine compounds inhibit cell growth and induce autophagy in human prostate cancer cells

**Figure 4:**
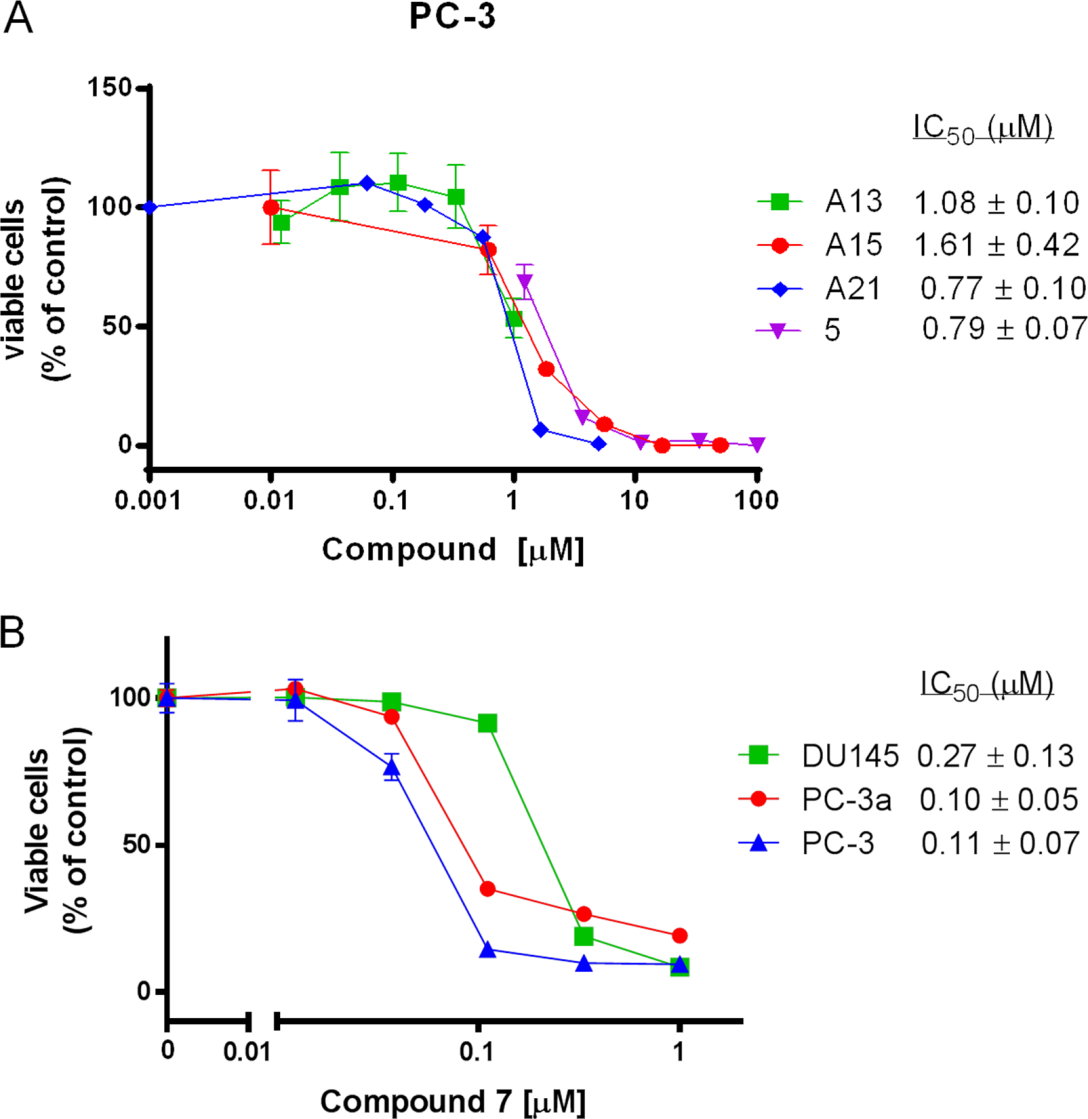
The growth inhibitory effects of several acridine compounds *in vitro* (**A**) Compounds A13, A15, A21, and 5 inhibit PC-3 prostate cancer cell growth in a dose-dependent manner. (**B**) The cytotoxicity of compound 7 against DU145, PC-3a and PC-3 cancer cell lines.

Three acridine analogues, A13, A15, and A21, were further assessed in cell-based assays. They exhibited potent inhibitory effects on the growth of human prostate cancer cell lines PC-3, PC-3a and DU145 (Table 1; Figure 4A), which have high levels of Bcl-2 family proteins. Moreover, A15 and A21 exhibited higher cytotoxicity in PC-3 and PC-3a than in DU145 cells, while A13 showed similar cytotoxicity to all three types.

**Table 1:** MTT-based cytotoxicity assay of acridine derivatives

Compound	Cytotoxicity in different cell lines (IC_50_, μM)		
	DU145	PC-3a	PC-3
A13	0.81 ± 0.51	0.68 ± 0.16	1.08 ± 0.10
A15	10.47 ± 1.11	2.33 ± 0.99	1.61 ± 0.42
A21	2.31 ± 0.39	0.80 ± 0.12	0.77 ± 0.10
1	8.15 ± 0.59	5.81 ± 1.16	6.04 ± 0.68
2	46.98 ± 7.39	29.20 ± 11.24	38.73 ± 5.46
3	> 100	82.66 ± 7.99	NT
4	6.33 ± 0.32	4.37 ± 0.68	4.43 ± 0.46
5	2.78 ± 0.79	0.74 ± 0.45	0.79 ± 0.07
6	> 100	> 100	> 100
7	0.27 ± 0.13	0.10 ± 0.05	0.11 ± 0.07
8	3.68 ± 0.55	1.64 ± 0.78	1.42 ± 0.73
9	6.24 ± 1.86	3.51 ± 1.15	2.93 ± 1.18
10	77.66 ± 24.17	36.09 ± 8.23	14.13 ± 0.71
11	> 100	> 100	> 100
12	45.44 ± 8.18	16.42 ± 2.56	21.56 ± 2.24
13	56.97 ± 14.45	53.86 ± 3.66	52.39 ± 10.36
14	> 100	> 100	> 100
15	> 100	> 100	> 100
16	35.99 ± 13.97	38.69 ± 20.42	44.96 ± 20.17
17	52.96 ± 14.71	> 100	29.49 ± 28.36

DU145, PC-3, and PC-3a: Human prostate cancer cell lines. Values are averages of at least two independent experiments. NT: Not tested.

**Figure 5:**
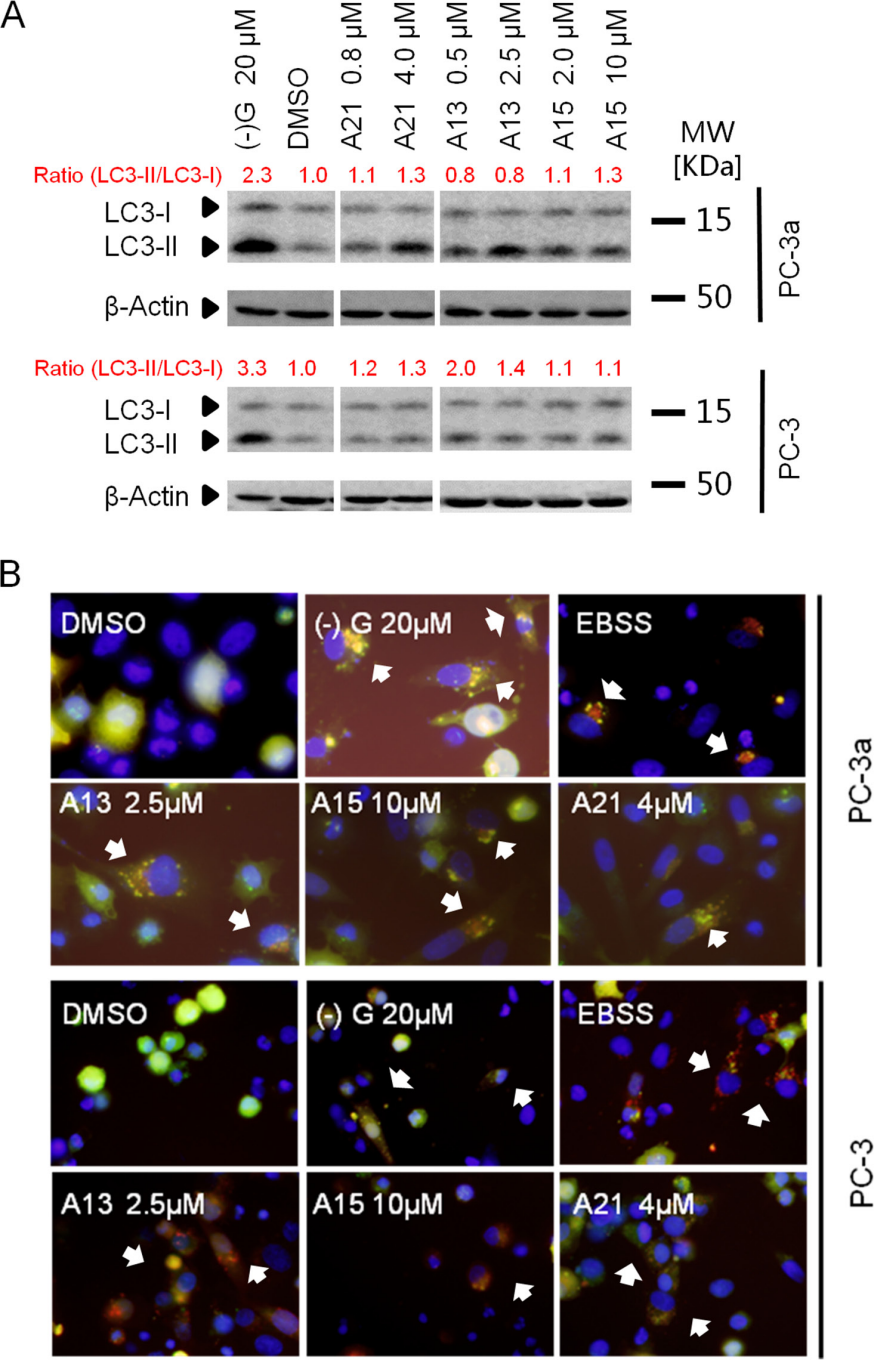
Acridine compounds induce autophagy in prostate cancer cells (**A**) LC3-II conversion in western blot analysis indicated the activity of autophagy. Cells were treated with DMSO or compounds for 24 h, and then lysed and processed for western blot analysis of LC3. (–)G and EBSS were used as positive controls. (**B**) RFP-GFP-LC3 puncta (indicated by white arrows) in cells after treatment suggested the induction of autophagy. Yellow puncta indicate autophagosome (overlay of red and green fluorescence); red puncta indicate autolysosome (due to green fluorescence quenching at the acidic condition). Cells expressing RFP-GFP-LC3 were treated with compounds A13, A15 or A21 at indicated concentration for 24 h.

Next, we tested LC3 conversion by the immunoblotting in PC-3 and PC-3a prostate cancer cells. Conversion of LC3-I to LC3-II is widely accepted as a marker of autophagy [34]. Increased level of LC3-II was observed in cells treated with A13, A15 or A21, demonstrating their abilities to induce autophagy (Figure 5A). Using a tandem fluorescent-tagged LC3, we monitored the autophagic flux levels in PC-3 and PC-3a cells. Because GFP (green fluorescent protein) signal is quenched at the low pH inside the lysosomes, while RFP (red fluorescent protein) is stable at low pH condition [35], this assay could detect autophagosomes (yellow puncta) and autolysosomes (red puncta) at the same time. As shown in Figure 5B, we observed many strong red puncta in PC-3a and PC-3 cells transfected with RFP-GFP-LC3 and treated with compounds A13, A15, or A21 for 24 h. This result demonstrated the appearance of autolysosomes and the activation of autophagic flow in the cells. Based on the results of two cellular assays, we established the activation of autophagic process in PC-3 and PC-3a treated with compound A13, A15 or A21. These results are consistent with our design strategy that the compounds disrupting Bcl-xL-Beclin 1 interaction could induce autophagy.

### Structure-activity relationship (SAR) analysis of A21 derivatives

**Figure 6:**
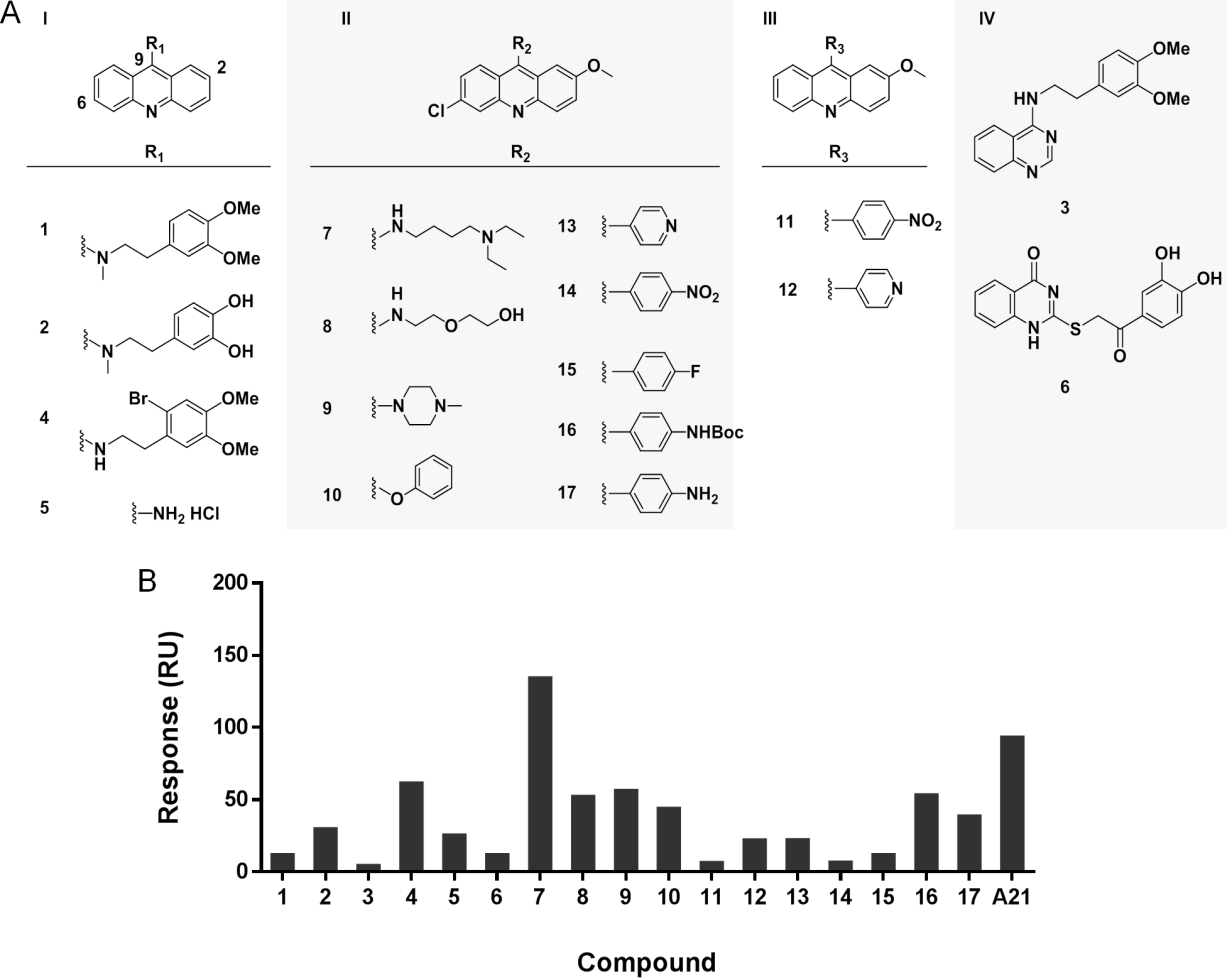
Analysis of structure-activity relationship of A21 derivatives (**A**) The chemical structures of acridine derivatives. (**B**) The maximal binding levels of acridine analogues to Bcl-xL protein at a concentration of 10 μM were tested using SPR. The compounds showed different binding ability to Bcl-xL protein.

To test the activity of acridine structure and gain a primary SAR, we built a set of seventeen A21 analogues. According to their structures, these compounds were divided into four groups (Figure 6A). Group I (1, 2, 4 and 5) includes compounds with only one substitution at 9 position of the acridine ring; group II (7–10 and 13–17) contains 6-chloro-2-methoxyacridines with different substitutions at 9 position; group III (11 and 12) consists of 2-methoxyacridines with different substitutions at 9 position; and the acridine rings of compounds in group IV (3 and 6) were replaced by other heterocycles.

The binding affinity of these acridine analogues to Bcl-xL protein was determined using the SPR assay. Figure 6B showed that only compound 7 had higher binding level than original hit A21 at the concentration of 10 μM, while compounds 1, 3, 6, 11, 14 and 15 had almost no binding to Bcl-xL. The results suggest that the acridine scaffold is essential for compounds to maintain their binding to target, as acridine-lacking group IV has the worst performance in this assay. Moreover, substituent group at the 9 position of acridine ring plays a significant role for the interaction between Bcl-xL and compound, as shown in different performance of compounds 1, 2, 4, 5 and compounds 7, 8, 9, 10. To be specific, the amino substituent is better than alkyl group at 9 position, and secondary amine is preferred to tertiary amine for a higher affinity. In addition, we found the chloro group at 6 position has no effect on the binding ability, when compared the results of compounds 11 to 14, as well as 12 to 13.

We also tested the cytotoxicity of the 17 compounds in human prostate cancer cell lines by MTT assay (Table 1). Compound 7 exhibited the most potent cytotoxicity against all three cell lines with IC_50_ values less than 0.5 μM (Figure 4B). Compound 5 also showed potent cytotoxicity with IC_50_ in between 0.5 μM and 5 μM. However, compounds 3, 6, 11, 14 and 15 showed no cytotoxicity in this assay (IC_50_> 100 μM). Notably, compounds 5 and 7 displayed higher cytotoxicity in PC-3 and PC-3a cells than in DU145 cells (3–5 fold difference in IC_50_ value). In summary, cytotoxicity of most compounds correlate with their binding abilities to Bcl-xL protein, which indicate that these compounds may reduce the cell viability *via* disrupting an oncogenic pathway involving Bcl-2 family proteins.

### Compound 7 selectively binds to Bcl-xL and induces autophagy

**Figure 7:**
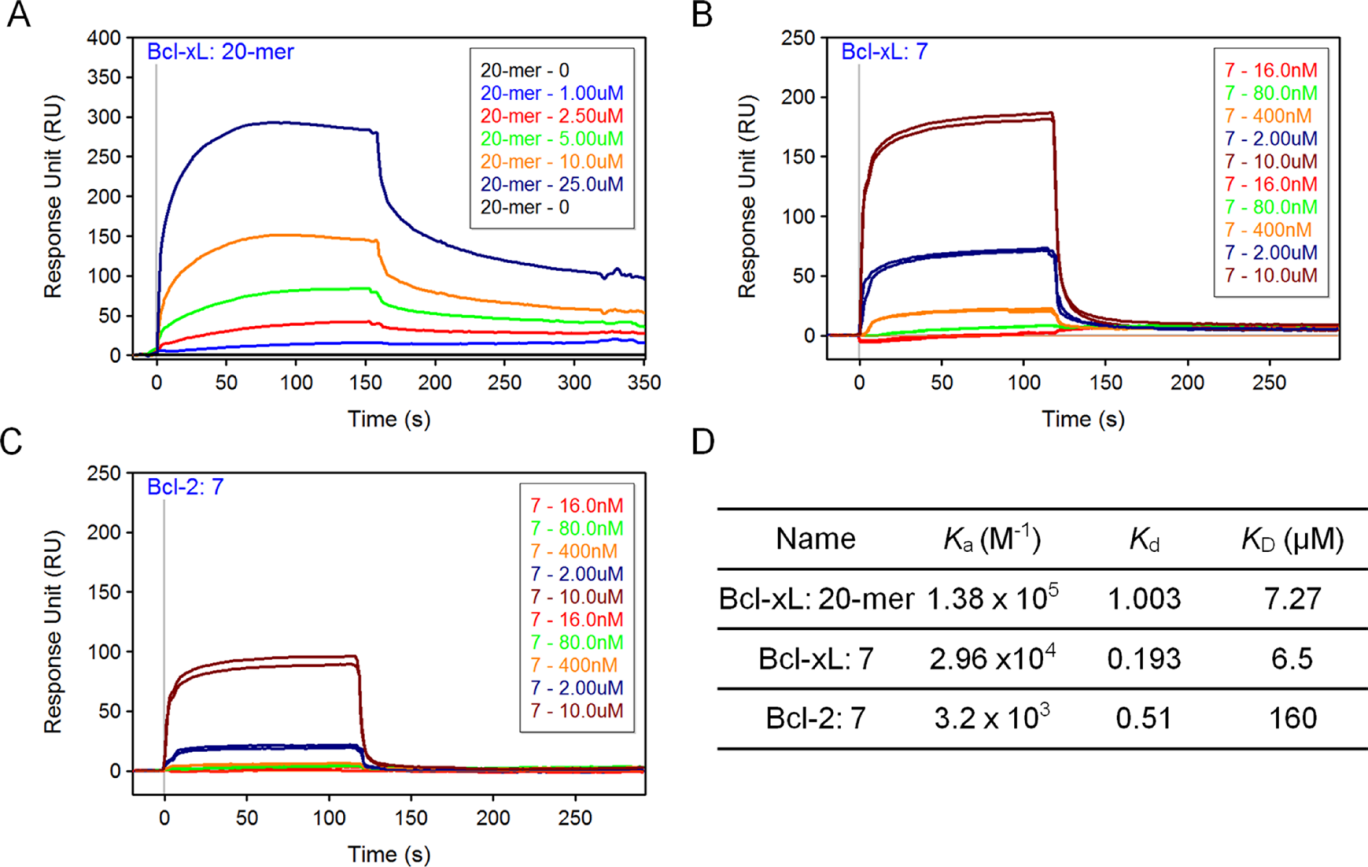
SPR analysis of compound 7 (**A, B, C**) SPR binding curves, or sensorgrams, for Beclin 20-mer peptide to immobilized Bcl-xL (A), compound 7 to Bcl-xL (B) and compound 7 to Bcl-2 (C). Sensorgrams representing direct binding kinetics for analytes are shown in response unit (RU) as a function of time (second) with increasing concentration (0.016–10 μM for 7; 1–25 μM for 20-mer). (**D**) The association rate (*K*_a_), dissociation rate (*K*_d_) and equilibrium dissociation constant (*K*_D_) were listed. *K*_D_ was determined by *K*_d_/*K*_a_.

From the library of A21 derivatives, compound 7 was identified with potent binding ability to Bcl-xL and cytotoxicity against prostate cancer cells (Figure 4B; Figure 6B). To gain a further insight into the interactions of compound 7 to Bcl-xL/2 protein, we used the SPR assay to test the binding affinity and kinetics. We found Beclin 1 20-mer peptide and compound 7 have comparable binding affinity to immobilized Bcl-xL protein (*K*_D_ = 7.3 μM and *K*_D_ = 6.5 μM, respectively; Figure 7A-7B). We also compared the binding behaviors of compound 7 to the Bcl-xL with that to Bcl-2 protein. It showed that compound 7 has similar dissociated rates to these two proteins, but it associates faster to Bcl-xL than to Bcl-2, thus having a lower affinity to Bcl-2 protein (*K*_D_ = 160.0 μM; Figure 7C–7D).

**Figure 8:**
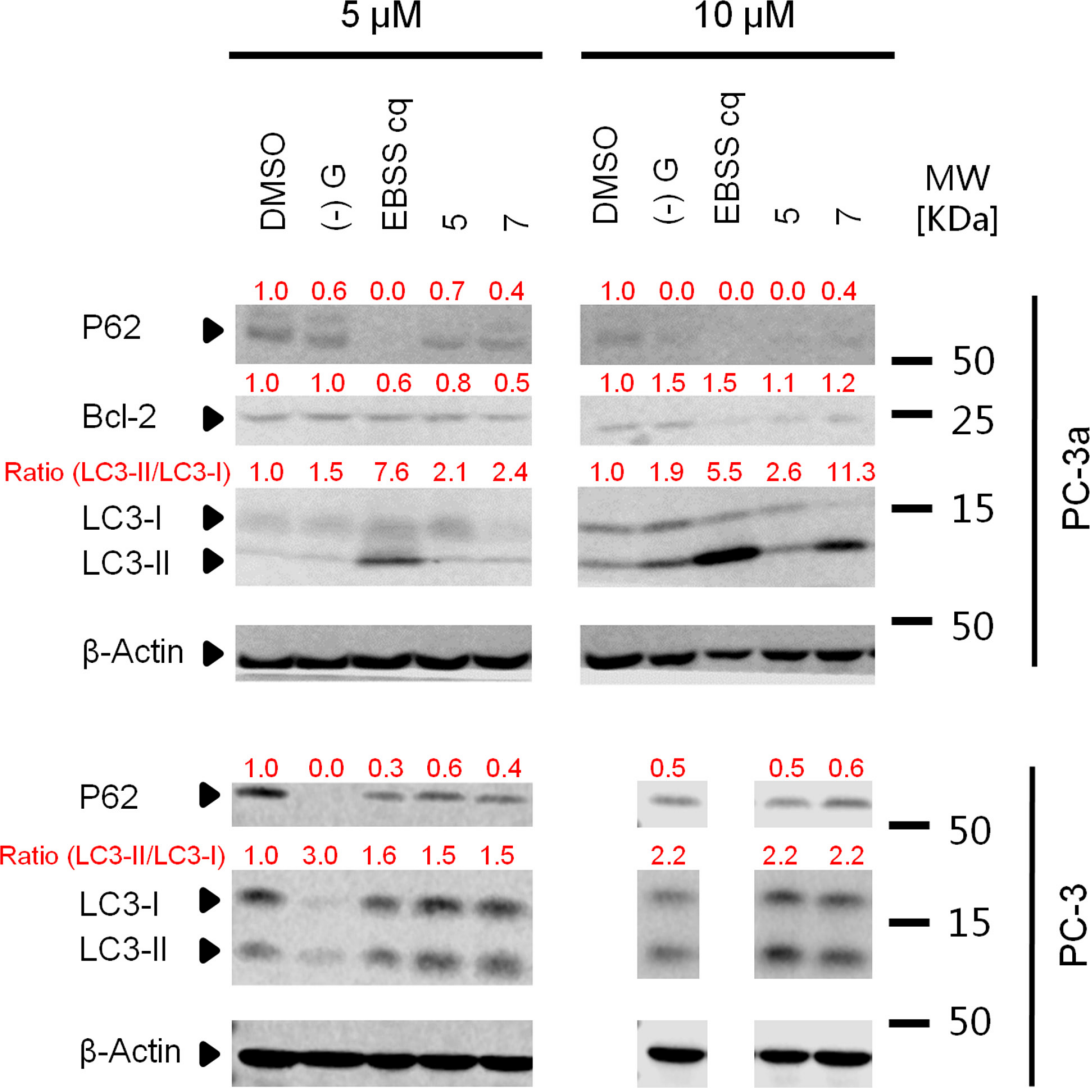
Acridine compounds induce autophagy in PC-3 and PC-3a cancer cells LC3-II conversion in western blot analysis indicated the activity of autophagy. Cells were treated with DMSO or compounds for 24 h, and then lysed and processed for western blot analysis of LC3. (–)G and EBSS were used as positive controls.

We next tested the effects of compound 7 on the autophagy pathway using western blotting. In addition to LC3 conversion, SQSTM1/P62 turnover can also be an important marker for assessing the autophagic flux [34]. Compound 7 triggered the conversion of LC3-I to LC3-II and the degradation of P62 in PC-3 and PC-3a cells, indicating its ability to induce autophagy (Figure 8).

## DISCUSSION

Here, we reported the efforts to screen and discover small molecule compounds mimicking the Beclin 1 protein, a BH3-only protein and key inducer of autophagy. In FP-based HTS with 50,316 compounds, we identified 58 initial hit compounds that block the interaction of Beclin 1 20-mer to Bcl-xL protein. After structure similarity clustering, three acridine analogues, A13, A15 and A21, were subjected to further analysis. Their binding affinities were reconfirmed in both FP and SPR assay (A13, *K*_i_= 0.84 μM; A15, *K*_i_ = 28.04 μM; A21, *K*_i_ = 13.15 μM). They showed potent anti-proliferative activity against prostate cancer cells (PC-3, PC-3a and DU145). They activated autophagic flux in PC-3 and PC-3a, determined by LC3 conversion and LC3 puncta assay. These acridine analogues represent a promising starting point for our SAR study. Thus, a small library of 17 acridine analogues has been set up and tested. Compound 7 was identified to bind to Bcl-xL (*K*_D_ = 6.5 μM), inhibit cell growth (IC_50_ < 0.5 μM against all three cells), and induce autophagy in PC-3 and PC-3a prostate cancer cells. Notably, compound 7 has selectivity for Bcl-xL protein (about 25-fold over Bcl-2). These results validated our design hypothesis that Beclin 1 mimetics, compounds disrupting Bcl-xL-Beclin 1 interaction, could induce autophagy and inhibit cell growth in apoptosis-deficient cancer cells.

Autophagy and apoptosis are two prominent self-destructive processes that control the fate of cells. Generally the cell switches between the two responses in a mutually exclusive manner [36]. However, in some cases, autophagy may help to active apoptosis, and excessively autophagy could even lead to autophagic cell death [4]. Early study showed that autophagy-associated cell death can be seen as a backup cell death mechanism when apoptosis fails [8]. Bcl-2 family proteins have been implicated in the regulation of both autophagy and apoptosis [27, 28, 37, 38]. The anti-apoptotic proteins from this family, such as Bcl-2, Bcl-xL or Mcl-1, could inhibit autophagy by binding to Beclin 1 [36]. Some pro-apoptotic BH3 only proteins, such as Bad, Noxa, Puma, could active autophagy by blocking the interaction between Beclin 1 and anti-apoptotic Bcl-2 family members. Moreover the C-terminus of cleaved Beclin 1 (Beclin 1-C) have a apoptosis-promoting function [39]. The Bcl-2/xL-Beclin 1 interaction represents a intriguing molecular mechanism between the autophagy and apoptosis. We here focused on the Bcl-xL-Beclin 1 complex, and explored Beclin 1-mimicking agents that disrupt the complex and promote autophagy. However, the effects of our compounds on the apoptotic pathway are still uncertain and warrant further investigation.

Given that deficient autophagy is linked with a wide range of human diseases, many investigations are devoted to identify autophagy inducers [19, 35, 40, 41]. Several available FDA-approved drugs have been repurposed for use in clinical indications, because of their autophagy-inducing activity [10, 42]. However, those drugs possess autophagy-inducing effects as well as other non-autophagy-inducing activities (side effects). The discovery and optimization of novel scaffolds, for maximal specificity and minimal side effect, is a major challenge in this field. The acridine structures we found in this study provided novel templates for further medicinal chemistry optimization. The primary SAR analysis of 17 acridine analogues provided information for further structure-based modifications and lead optimization.

These Beclin 1 mimetics might be promising tools for understanding the roles of Bcl-xL protein, Beclin 1, and even the multifaceted roles of autophagy in cancer development and drug resistance. In MTT-based cytotoxicity assay, PC-3a and PC-3 cells seem to be more responsive to the compounds than DU145 cells, potentially due to higher levels of Bcl-xL/2. As those compounds have been proven to bind to Bcl-xL (7 has selectivity for Bcl-xL), PC-3a and PC-3 cells are more likely to rely upon Bcl-xL for its sustained growth.

Taken together, through our HTS effort and following structural, biochemical, and cellular evaluations, we have identified four potential Beclin 1 mimetics, A13, A15, A21 and 7. They have high binding affinity to Bcl-xL, kill prostate cancer cells *via* disrupting a Bcl-xL-related oncogenic pathway and activate autophagic flux in prostate cancer cells. These Beclin 1 mimetics represent new templates warranting further medicinal chemistry optimization. Furthermore, these compounds will be good tools for exploring the roles of Bcl-xL in malignant tumor cells and investigating the potential of autophagy to kill cancer cells. Future plan includes design and synthesis of novel acridine-based Beclin 1 mimetics for high potency and selectivity. Moreover, future experiments will focus on exploring target effectiveness, target specificity and mechanism of action studies with the ultimate goal of developing novel small molecule Beclin 1 mimetics modulating autophagy for clinical benefits.

## MATERIALS AND METHODS

### Cell culture and reagents

Human prostate cancer cell lines PC-3 and DU145, were purchased from American Type Culture Collection (ATCC) and used within 50 passages. PC-3a is a derivative of PC-3 that is resistant to chemo/radiotherapy, established in our lab and confirmed by genotyping. All cells were maintained in DMEM (Mediatech, Manassas, VA) supplemented with 10% fetal bovine serum (FBS) (Sigma-Aldrich, St. Louis, MO), 1% Glutamine (Mediatech, Manassas, VA), 1% antibiotics (Mediatech, Manassas, VA). Compounds for primary FP-based HTS screening were from Prestwick, Microsource, University of Kansas Center of Excellence in Chemical Methodologies & Library Development (KU CMLD, Lawrence, KS), and Chembridge libraries. Compounds for MTT and SPR validation experiment were purchased from Microsource (Gaylordsville, CT) and Hit2lead Company (San Diego, CA). Acridine analogues, compound 1–6, were from our in-house library (provided by Specialized Chemistry Center, University of Kansas, Lawrence, KS); compound 7–17 were synthesized as described in supplementary information. (–)-Gossypol was isolated from racemic gossypol as we previously described [8, 43]. ABT-263 and DMSO were purchased from Sigma. MTT-based cytotoxicity assay and western blot analysis was carried out according to our previous publications [44]. The primary antibodies used were LC3B (#2775) (Cell signaling Technology, Danvers, MA) and β-actin (A5441) (Sigma).

### Fluorescence polarization assay

All *N*′-fluorescein-labeled peptides were purchased from Kansas State University (Manhattan, KS). Beclin 1 8-mer, 10-mer, 12-mer, 20-mer and 26-mer peptides were designed in our lab. 16mer Bak (GQVGRQLAIIGDDINR) sequence was from published literature [29, 45]. Bcl-xL protein (25 kDa, > 90% pure based on SDS–PAGE) was expressed and purified in Protein Product Group, University of Kansas. FP assays were carried out as described in our previous publication [44, 46] with some modified. For the assay optimization and determination of equilibrium dissociation constant (*Kd*), purified proteins were serially diluted in binding buffer (20 mM HEPES, pH 7.4, 0.05% pluronic F-68), and added to 96-well assay plates (Corning 3915) (Corning, Corning, NY). Then, fluorescein-labeled peptide was added to each well with a final concentration of 50 nM and incubated at room temperature for 30 min. Anisotropy measurements were taken at room temperature using a BioTek Synergy H4 plate reader (Biotek, Winooski, VT) following the protocol recommended by the manufacture. The *Kd* was estimated by nonlinear regression to a one-site binding model using GraphPad Prism 5.0 (GraphPad, San Diego, CA). For competitive binding experiment, compound in serial dilutions (1 nM to 100 μM) was added to Bcl-xL-Beclin 1 20-mer complex in the assay buffer in a 96-well black microplate. The final concentrations of Bcl-xL and 20-mer were 2 μM and 50 nM, respectively. Compound solvent DMSO only was used as a negative control in the competition assay. After 2 h incubation, the plate was read and polarization was calculated.

### FP-based high-throughput screening

To form the Bcl-xL-Beclin 1 complex, Bcl-xL was incubated with Beclin 1 20-mer in binding buffer (20 mM HEPES, pH 7.4, 0.05% Pluronic F-68) for 5 min at 25°C. Forty microliter aliquots of the mixture were added to the 384-well plates containing the compounds (for a final concentration of 2.5 μM Bcl-xL, 400 nM 20-mer, and 40 μM compounds in the first screen; and a final concentration of 2.5 μM Bcl-xL, 300 nM 20-mer, and 30 μM compounds in the second screen) and further incubated at 25°C for 2 h. Then the plates were read by Envision (Perkin Elmer, Waltham, MA). The compounds with an inhibition of greater than plate median plus 3 standard deviation were selected as hits. The hits were selected from library stocks and reconfirmed in a concentration response FP assay (6 doses ranging from 5 μM to 160 μM or 8 doses ranging from 0.5 μM to 60 μM). The percent of inhibition was normalized such that the FP value of the protein-peptide complex with DMSO was defined as 0% inhibition, while the FP value obtained with the same concentration of the free fluorescein-labeled 20-mer alone was defined as 100% inhibition.

### Surface plasmon resonance assay

The surface plasmon resonance experiments were performed using a Biacore 3000 (GE Healthcare Pittsburgh, PA) equipped with a research-grade CM5 sensor chip. The ligand (Bcl-xL) was immobilized using amine-coupling chemistry. The surfaces of flow cells on a Biacore sensor chip CM5 were activated for 7 min with a 1:1 mixture of 0.1 M NHS (N-hydroxysuccinimide) and 0.1 M EDC (3-(*N*,*N*-dimethylamino) propyl-*N*-ethylcarbodiimide) at a flow rate of 5 μL/min. The ligand (Bcl-xL) at a concentration of 180 μg/mL in 10 mM sodium acetate, pH 4.5, was immobilized at a density of about 10000 RU on flow cell 2; the ligand (SUMO-Bcl-2, 37.5 kDa, > 90% pure based on SDS–PAGE) at a concentration of 44 μg/ml in 10 mM sodium acetate, pH 4.5, was immobilized at a density of 10000 RU on flow cell 3; flow cell 1 was left blank to serve as a reference surface. All the surfaces were blocked with a 7 min injection of 1 M ethanolamine, pH 8.0.

For kinetic analysis, a concentration series of analyte were injected over the flow cells at a flow rate of 60 μL/min at 20 °C. The complex was allowed to associate and dissociate for 120 and 180 s, respectively. For binding studies, analyte (or test compound) was injected over the two flow cells in duplicate at indicated concentration at a flow rate of 60 μL/min at 20 °C. The running buffer consisted of 20 mM HEPES, 150 mM NaCl, 3 mM EDTA, 1 mM DTT, 0.05% P20, 5% DMSO, pH 7.4. The complex was allowed to associate and dissociate for 120 and 180 s, respectively. The buffer samples containing a range of 4–6% DMSO were injected at the beginning, used for solvent correction. The surfaces were regenerated between binding cycles with a 5 s injection of 5 mM NaOH for compounds and 10 mM Glycine pH 2.5 for 20-mer.

All measurements were taken with flow cell 1 as a reference (i.e. sensorgram depicts “2–1 correction”) and corrected for DMSO bulk differences *via* the calibration curves. To obtain kinetic rate constants (*K*_a_ and *K*_d_), corrected response data were fit to a simple 1:1 interaction model using local fit (includes correction for mass transport limitations) available within Biaevaluation 3.1 software or Scrubber2 software. The equilibrium dissociation constant (*K*_D_) was determined by *K*_d_/*K*_a_.

### GFP-RFP-LC3 analysis

PC-3 and PC-3a cells were transfected with ptfLC3 plasmid using Lipofectamine 2000 (Invitrogen Life Technologies, Carlsbad, CA). ptfLC3 was a gift from Tamotsu Yoshimori (Addgene plasmid # 21074) [47], and is a tandem fluorescent-tagged LC3 with mRFP (mRFP, monomeric Red Fluorescent Protein) and EGFP (EGFP, Enhanced Green Fluorescent Protein). Twenty-four hours after transfection, cells were treated with compounds for 24 h, then fixed in 4% formaldehyde for 10 min. Cells were then washed three times with PBS, stained with DAPI (0.5 μg/ml in PBS) for 5 min and observed under a fluorescence microscope (OLYMPUS IX81, Tokyo, Japan) with X 40 lens.

## SUPPLEMENTARY MATERIALS FIGURE AND TABLES





## Abbreviations

HTS: high-throughput screening
FP: fluorescence polarization
KU CMLD: University of Kansas Center of Excellence in Chemical Methodologies & Library Development
CID: PubChem Chemical ID
SPR: surface plasmon resonance
RU: response units
SAR: structure-activity relationship
MTT: 3-(4,5dimethylthiazol-2-yl)-2,5-diphenyltetrazolium bromide
LC3: light chain 3
PAINS: pan-assay interference compounds
RFP: red fluorescent protein
GFP: green fluorescent protein.
