# Supplementary material for: Identification of novel small molecule Beclin 1 mimetics activating autophagy
Source: Oncotarget. 2017 May 18;8(31):51355–69. doi: 10.18632/oncotarget.17977 (PMC5584254; doi:10.18632/oncotarget.17977)
Supplement: Supplementary file 1 [file oncotarget-08-51355-s001.pdf]

Identification of novel small molecule Beclin 1 mimetics activating autophagy

Supplementary Materials

CHEMISTRY

All chemical reagents were purchased from commercial sources and used without further purification. Thin-layer chromatography (TLC) was conducted on silica gel GF-254 glass plates. ^{1}H NMR and ^{13}C NMR spectra were recorded on a Bruker Avance 400 (400 MHz) NMR spectrometer. ESI-MS was recorded on Agilent 1100 LC/MSD (70 ev) spectrometer. High resolution mass spectra (HRMS) were recorded on a Waters Q-Tof micro mass spectrometer.

General procedure I.*N*^{1}-(6-Chloro-2-methoxyacridin-9-yl)-*N*^{4},*N*^{4}-diethylbutane-1,4-diamine (7)

6,9-Dichloro-2-methoxyacridine (200 mg, 0.72 mmol) and phenol (1.7 g, 18mmol) were stirred at 100 ˚C for 1h before addition of 4-(diethylamino)butylamine (0.125 mL, 0.76 mmol). The reaction mixture was allowed to react at 100 ^{o}C for 3 h and then cooled at room temperature. The mixture was basified with 2N NaOH (40 mL) and extracted with ethyl acetate (3 × 50 mL). The organic layer was washed with brine, dried over Na_2SO_4, filtered and concentrated under reduced pressure. The residue was purified by reverse phase chromatography (CH_3CN: H_2O: NH_4OH) to give the pure product **7** as yellow oil, yield 103 mg, 37%.^{1}H NMR (400 MHz, Chloroform-*d*) δ 7.99 (d, *J* = 2.1 Hz, 1H), 7.94 (d, *J* = 9.3 Hz, 1H), 7.93 (d, *J* = 9.4 Hz, 1H), 7.35 (dd, *J* = 9.4, 2.7 Hz, 1H), 7.20 (d, *J* = 2.1 Hz, 1H), 7.18 (d, *J* = 2.2 Hz, 1H), 7.17 (d, *J* = 2.7 Hz, 1H), 5.18 (s, 1H), 3.88 (s, 3H), 3.64 (t, *J* = 6.8 Hz, 2H), 2.47 (q, *J* = 7.2 Hz, 4H), 2.41 (t, *J* = 7.2 Hz, 2H), 1.72 (p, *J* = 6.9 Hz, 2H), 1.55 (p, *J* = 7.3 Hz, 2H), 0.95 (t, *J* = 7.2 Hz, 6H); ^{13}C NMR (101 MHz, Chloroform-*d*) δ 155.73, 149.99, 148.38, 146.81, 134.67, 131.36, 128.12, 124.31, 124.30, 124.14, 117.72, 115.68, 99.72, 55.54, 52.18, 50.62, 46.79, 29.70, 24.89, 11.43; IR ν_{max} (cm^{-1}) 3271, 2950, 2828, 1562, 1435, 1236, 823; HRMS calcd for $C_{22}H_{29}ClN_3O$ [M+H]$^+$ 386.19, found 386.20.

2-(2-((6-Chloro-2-methoxyacridin-9-yl)amino)ethoxy)ethanol (8)

8 was prepared from 6,9-dichloro-2-methyoxyacridine and 2-(2-aminoethoxy)ethanol according to general procedure I. Yield 249 mg, 99%, m.p. 142-145.^{1}H NMR (400 MHz, Chloroform-*d*) δ 8.08 (d, *J* = 2.1 Hz, 1H), 8.04 (d, *J* = 9.1 Hz, 1H), 8.01 (d, *J* = 9.4 Hz, 1H), 7.42 (dd, *J* = 9.4, 2.7 Hz, 1H), 7.31 (dd, *J* = 9.2, 2.1 Hz, 1H), 7.25 (s, 1H), 3.96 (s, 3H), 3.84 (ddd, *J* = 9.2, 5.4, 4.2 Hz, 4H), 3.68–3.59 (m, 4H); ^{13}C NMR (126 MHz, Chloroform-*d*) δ 156.27, 149.67, 148.21, 146.80, 134.90, 131.42, 128.19, 124.91, 124.86, 124.28, 118.98, 116.93, 99.19, 72.58, 70.23, 61.85, 55.63, 49.99; IR ν_{max} (cm^{-1}) 3323, 3097, 2900, 2837, 1562, 1431, 1228, 1135, 827, 768; HRMS calcd for $C_{18}H_{20}ClN_2O_3$ [M+H]$^+$ 347.11, found 347.11.

6-Chloro-2-methoxy-9-(4-methylpiperazin-1-yl)acridine (9)

9 was prepared from 6,9-dichloro-2-methyoxyacridine and 1-methylpiperazine according to general procedure I. Yield 115 mg, 47%, m.p. 135-138. ^{1}H NMR (400 MHz, Chloroform-*d*) δ 8.31 (d, *J* = 9.3 Hz, 1H), 8.16 (d, *J* = 1.9 Hz, 1H), 8.07 (d, *J* = 9.4 Hz, 1H), 7.52 (d, *J* = 2.8 Hz, 1H), 7.45 (dd, *J* = 9.4, 2.8 Hz, 1H), 7.40 (dd, *J* = 9.3, 2.1 Hz, 1H), 3.98 (s, 3H), 3.66–3.59 (m, 4H), 2.79–2.72 (m, 4H), 2.50 (s, 3H); ^{13}C NMR (126 MHz, Chloroform-*d*) δ 156.97, 152.28, 148.89, 148.20, 134.73, 131.87, 128.72, 126.33, 125.87, 125.68, 125.44, 123.46, 100.38, 56.56, 55.73, 52.08, 46.83; IR ν_{max} (cm^{-1}) 2937, 2831, 2787, 1420, 1225, 802, 770; HRMS calcd for $C_{19}H_{21}ClN_3O$ [M+H]$^+$ 342.13, found 342.13.

6-Chloro-2-methoxy-9-phenoxyacridine(10)

10 was the by-product separated from the above preparation of compound **9**. Yield 36 mg, 15%. ^{1}H NMR (400 MHz, Chloroform-*d*) δ 8.23 (dd, *J* = 2.0, 0.5 Hz, 1H), 8.16–8.08 (m, 1H), 7.98 (dd, *J* = 9.2, 0.5 Hz, 1H), 7.48 (dd, *J* = 9.5, 2.8 Hz, 1H), 7.37 (dd, *J* = 9.2, 2.0 Hz, 1H), 7.32–7.26 (m, 2H), 7.16 (d, *J* = 2.8 Hz, 1H), 7.10–7.02 (m, 1H), 6.86 (t, *J* = 1.6 Hz, 1H), 6.85 (dd, *J* = 2.0, 0.9 Hz, 1H), 3.81 (s, 3H); ^{13}C NMR (126 MHz, Chloroform-*d*) δ 159.13, 157.63, 153.60, 148.79, 148.64, 135.56, 131.40, 130.17, 128.29, 127.41, 126.63, 123.87, 122.93, 121.33, 119.00, 115.72, 97.85, 55.72; IR ν_{max} (cm^{-1}) 3044, 2919, 2850, 1630, 1471, 1206, 755; HRMS calcd for $C_{20}H_{15}ClNO_2$ [M+H]$^+$ 336.07, found 336.09.

2-Methoxy-9-(4-nitrophenyl)acridine (11)

11 was prepared from 6,9-dichloro-2-methyoxyacridine and 4-nitrophenylboronic acid pinacol ester according to general procedure II. But the reaction temperature is 150°C, and the reaction time is 30 minutes. m.p. 244-246.^{1}H NMR (400 MHz, Chloroform-*d*) δ

8.54–8.46 (m, 2H), 8.27 (dt, *J* = 8.8, 1.0 Hz, 1H), 8.20 (d, *J* = 9.5 Hz, 1H), 7.74 (ddd, *J* = 8.7, 6.0, 1.9 Hz, 1H), 7.69–7.63 (m, 2H), 7.52–7.42 (m, 3H), 6.64 (d, *J* = 2.7 Hz, 1H), 3.75 (s, 3H); ^{13}C NMR (126 MHz, Chloroform-*d*) δ 157.69, 148.10, 147.12, 146.07, 143.80, 141.49, 131.73, 131.67, 130.07, 129.19, 126.71, 125.49, 125.41, 125.36, 124.84, 124.13, 101.00, 55.57; IR ν_{max} (cm^{-1}) 3031, 2924, 2852, 1513, 1346, 1226, 821, 748; HRMS calcd for $C_{20}H_{15}N_2O_3$ [M+H]$^+$ 331.1011, found 331.1086.

2-Methoxy-9-(pyridin-4-yl)acridine (12)

12 was prepared from 6,9-Dichloro-2-methyoxyacridine and 4-pyridinylboronic acid according to general procedure II. But the reaction temperature is 150°C, and the reaction time is 10 minutes.m.p. 170-174.^{1}H NMR (400 MHz, Chloroform-*d*) δ 8.94–8.87 (m, 2H), 8.26 (d, *J* = 8.8 Hz, 1H), 8.19 (d, *J* = 9.5 Hz, 1H), 7.78–7.69 (m, 1H), 7.57–7.39 (m, 5H), 6.69 (d, *J* = 2.7 Hz, 1H), 3.75 (s, 3H); ^{13}C NMR (126 MHz, Chloroform-*d*) δ 157.60, 150.48, 147.13, 146.07, 145.16, 141.01, 131.66, 130.01, 129.16, 126.60, 125.49, 125.47, 125.14, 124.55, 101.07, 55.56; IR ν_{max} (cm^{-1}) 3041, 2922, 2852, 2048, 1229, 820, 755, 746; HRMS calcd for $C_{19}H_{15}N_2O$ [M+H]$^+$ 287.1118, found 287.1188.

General procedure II.6-Chloro-2-methoxy-9-(pyridin-4-yl)acridine (13)

To a solution of 6,9-dichloro-2-methyoxyacridine (100 mg, 0.36 mmol) in 4mL of DMF/H_2O (4:1) was added 4-pyridinylboronic acid (44.2 mg, 0.36 mmol). The mixture was degassed with argon. Then bis(triphenylphosphine) palladium(II) dichloride ($PdCl_2(PPh_3)_2$)(50 mg, 0.072 mmol) and potassium carbonate(100 mg, 0.72 mmol) was added. The mixture was microwaved at 100°C for 5 minutes. After cooled at room temperature, the mixture was partitioned in EtOAc (80 mL) and H_2O (50 mL). The organic layer was separated, washed with brine, dried over Na_2SO_4 and concentrated under reduced pressure. The residue was purified by reverse phase chromatography at stand base condition (CH_3CN: H_2O: NH_4OH) to give the pure product **13**, yield 38%, m.p. 232-235. ^{1}H NMR (400 MHz, Chloroform-*d*) δ 8.94–8.88 (m, 2H), 8.26 (d, *J* = 1.6 Hz, 1H), 8.16 (d, *J* = 9.5 Hz, 1H), 7.50 (dd, *J* = 9.5, 2.8 Hz, 1H), 7.47 (d, *J* = 9.2 Hz, 1H), 7.44–7.37 (m, 2H), 7.38 (dd, *J* = 9.3, 2.1 Hz, 1H), 6.67 (d, *J* = 2.7 Hz, 1H), 3.75 (s, 3H); ^{13}C NMR (126 MHz, Chloroform-*d*) δ 157.84, 150.59, 147.09, 146.65, 144.64, 141.28, 135.16, 131.60, 128.45, 127.84, 126.90, 126.08, 125.32, 125.19, 122.92, 101.12, 55.60; IR ν_{max} (cm^{-1})3036, 2922, 2852, 2044, 1628, 1421, 1233, 836, 776; HRMS calcd for $C_{19}H_{14}ClN_2O$ [M+H]$^+$ 321.0723, found 321.0798.

6-Chloro-2-methoxy-9-(4-nitrophenyl)acridine (14)

14 was prepared from 6,9-dichloro-2-methyoxyacridine and 4-nitrophenylboronic acid pinacol ester according to general procedure II. Yield 17%, m.p. 233-236. ^{1}H NMR (400 MHz, Chloroform-*d*) δ 8.51 (d, *J* = 8.7 Hz, 2H), 8.26 (d, *J* = 1.8 Hz, 1H), 8.17 (d, *J* = 9.5 Hz, 1H), 7.65 (d, *J* = 8.7 Hz, 2H), 7.51 (dd, *J* = 9.5, 2.7 Hz, 1H), 7.46–7.34 (m, 2H), 6.62 (d, *J* = 2.7 Hz, 1H), 3.75 (s, 3H); ^{13}C NMR (126 MHz, Chloroform-*d*) δ 157.94, 148.25, 147.10, 146.68, 143.23, 141.77, 135.20, 131.66, 131.58, 128.53, 127.95, 126.79, 126.09, 125.48, 124.25, 123.22, 101.06, 55.62; IR ν_{max} (cm^{-1}) 2923, 2852, 1508, 1342, 1230, 827, 736; HRMS calcd for $C_{20}H_{14}ClN_2O_3$ [M+H]$^+$ 365.0608, found 365.0683.

6-Chloro-9-(4-fluorophenyl)-2-methoxyacridine (15)

15 was prepared from 6,9-dichloro-2-methyoxyacridine and 4-fluorophenylboronic acid pinacol ester according to general procedure II. Yield 33%, m.p. 185-187. ^{1}H NMR (400 MHz, Chloroform-*d*) δ 8.24–8.21 (m, 1H), 8.13 (d, *J* = 9.4 Hz, 1H), 7.54 (d, *J* = 9.2 Hz, 1H), 7.48 (dd, *J* = 9.4, 2.8 Hz, 1H), 7.40 (ddd, *J* = 8.0, 5.2, 2.4 Hz, 2H), 7.37–7.29 (m, 3H), 6.76 (d, *J* = 2.7 Hz, 1H), 3.75 (s, 3H); ^{13}C NMR (126 MHz, Chloroform-*d*) δ 163.94, 161.96, 157.46, 147.26, 146.72, 143.82, 134.98, 132.15, 132.08, 131.88, 131.85, 131.41, 128.26, 127.61, 127.31, 126.25, 125.79, 124.00, 116.20, 116.03, 101.83, 55.52; IR ν_{max} (cm^{-1}) 3043, 2954, 2925, 2825, 1629,1421, 1229, 801; HRMS calcd for $C_{20}H_{14}ClFNO$ [M+H]$^+$ 338.0673, found 338.0749.

tert-Butyl (4-(6-chloro-2-methoxyacridin-9-yl) phenyl)carbamate (16)

16 was prepared from 6,9-dichloro-2-methyoxyacridine and 4-(N-Boc-amino)phenylboronic acid pinacol ester according to general procedure II. Yield 43%, m.p.175-179. ^{1}H NMR (400 MHz, Chloroform-*d*) δ 8.24–8.19 (m, 1H), 8.12 (d, *J* = 9.4 Hz, 1H), 7.66–7.58 (m, 3H), 7.46 (dd, *J* = 9.4, 2.8 Hz, 1H), 7.39–7.34 (m, 2H), 7.32 (dd, *J* = 9.3, 2.1 Hz, 1H), 6.87 (d, *J* = 2.7 Hz, 1H), 6.77 (s, 1H), 3.75 (s, 3H), 1.58 (s, 9H); ^{13}C NMR (126 MHz, Chloroform-*d*) δ 157.30, 152.90, 147.30, 146.72, 144.75, 138.88, 134.89, 131.27, 131.15, 130.27, 128.12, 127.98, 127.03, 126.29, 125.74, 124.11, 118.61, 102.12, 81.16, 55.57, 28.51; IR ν_{max} (cm^{-1}) 2974, 2932, 1711, 1520, 1228, 1154, 812, 728; HRMS calcd for $C_{25}H_{24}ClN_2O_3$ [M+H]$^+$ 435.142, found 435.1496.

4-(6-Chloro-2-methoxyacridin-9-yl)aniline (17)

A solution of 16 (35 mg, 0.08 mmol) in 5 mL of TFA/DCM (1:4) was stirred overnight at room temperature until no 16 was observed by TLC. The reaction solution was concentrated in vacuo and purified by silica gel chromatography (5% NH_4OH: MeOH: DCM) to give pure product 17.Yield 95%, m.p.207-211. ^{1}H NMR (400 MHz, Chloroform-*d*) δ 8.24 (d, *J* = 1.8 Hz, 1H), 8.15 (d, *J* = 9.4 Hz, 1H), 7.71 (d, *J* = 9.3 Hz, 1H), 7.47 (dd, *J* = 9.4, 2.8 Hz, 1H), 7.32 (dd, *J* = 9.3, 2.1 Hz, 1H), 7.21 (q, *J* = 4.4

Hz, 1H), 7.21 (d, J = 8.5 Hz, 1H), 6.96 (d, J = 2.7 Hz, 1H), 6.91 (d, J = 8.4 Hz, 2H), 3.76 (s, 3H); ^{13}C NMR (101 MHz, Chloroform-d) δ 157.19, 146.85, 146.26, 135.18, 131.53, 130.74, 128.34, 127.62, 126.87, 126.57, 125.86, 125.34, 124.31, 115.22, 102.56, 55.54; IR ν_{max} (cm^{-1}) 3446, 3303, 3182, 1630, 1608, 1418, 1229, 811; HRMS calcd for $C_{20}H_{16}ClN_2O$ $[M+H]^+$ 335.0862, found 335.0938.

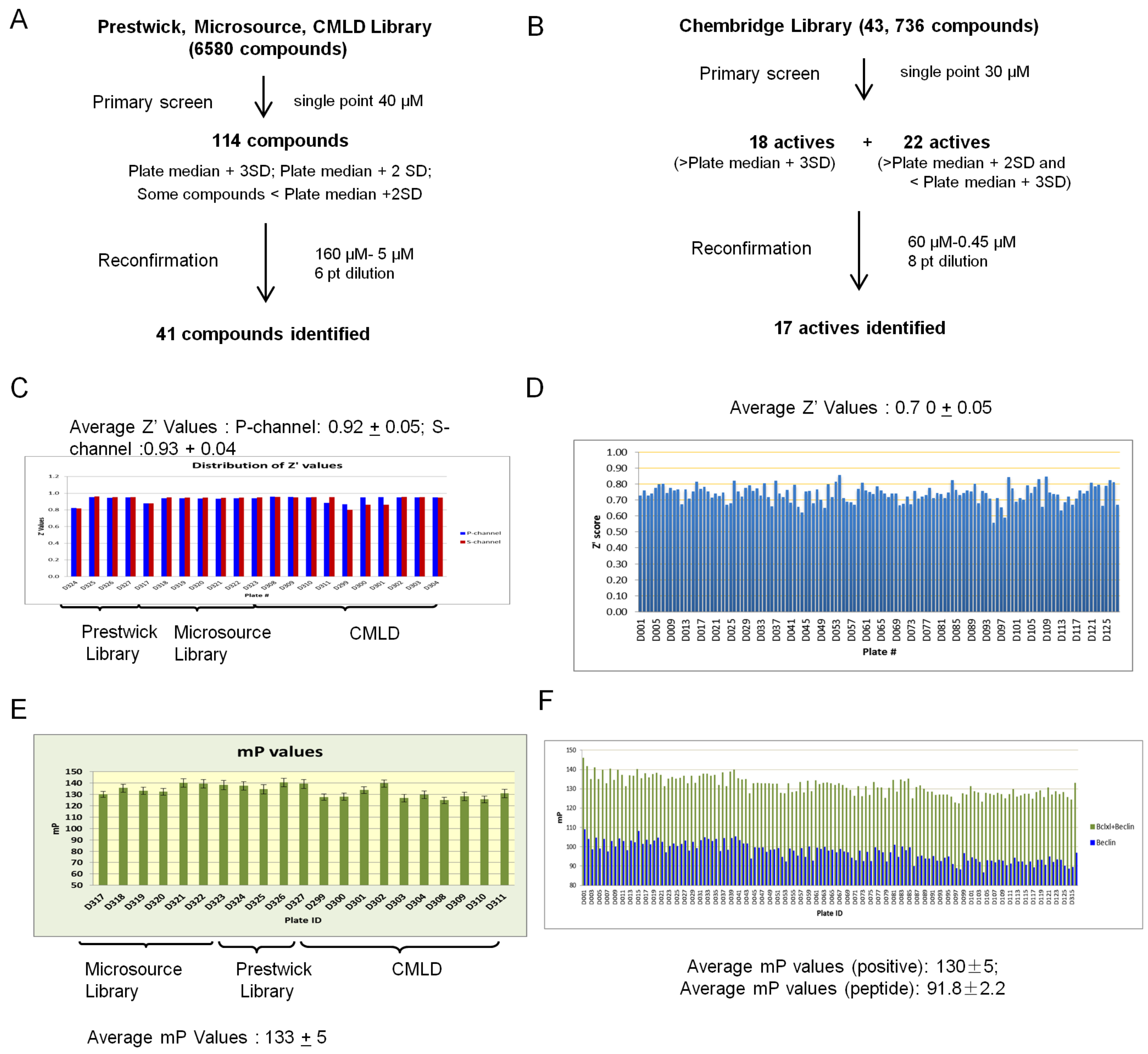

Supplementary Figure 1: FP-based high-throughput screening. HTS was performed with ~50000 compounds from Prestwick, Microsource, CMLD and Chembridge libraries. The four libraries were divided into two groups and screened separately. (**A, B**) High-throughput screening cascades of first screen (A) and second screen (B). (**C, D**) Distribution of Z′ values of first screen (C) and second screen (D). The average Z′ value is 0.82 ± 0.05, calculated using the same method in previous publication. (**E, F**) Distribution of mP values of first screen (E) and second screen (F).

Supplementary Table 1: Summary of screen libraries and actives

	Validation Library	Compounds	Hits	Hit rate
First Screen	Prestwick<br>Microsource<br>CMLD	6580	41	0.623%
Second Screen	Chembridge	43736	17	0.038%
Total		50316	58	0.115%

Supplementary Table 2: 41 hits from Microsource library, Prestwick library and CMLD library, with name, CID number, code, percentage of inhibition at 40 μM from primary screen, and dose-response reconfirmation data from secondary screen. See Supplementary_Table_2.

Supplementary Table 3: 17 hits from Chembridge library, with CID number, code, percentage of inhibition at 30 μM from primary screen, and dose-response reconfirmation data from secondary screen

	Plate ID	Coordinates	Code	CID	Primary Screen		Dose-response (% Inhibition)							
					mP	% Inhibition	60 μM	30 μM	15 μM	7.5 μM	3.75 μM	1.875 μM	0.975 μM	0.45 μM
1	D017	L10	B01	464050	86.7	36.8	79.7	67.5	52.5	33.5	20.2	12.5	4.2	2.3
2	D025	E17	B02	672470	62.4	53.5	72.2	68.5	66.0	54.8	44.1	28.7	15.5	6.6
3	D006	J14	B03	748380	74.7	46.0	67.8	60.9	56.0	54.4	45.1	38.0	31.9	18.0
4	D040	F18	B04	2927046	83.3	39.9	62.7	50.3	44.3	33.8	18.7	11.6	7.2	5.2
5	D002	B10	B05	137753	102.5	26.9	58.6	49.5	37.0	22.4	14.9	8.2	2.1	2.0
6	D021	M3	B06	335513	110.6	18.7	57.3	35.8	4.7	4.5	2.6	1.3	3.5	0.7
7	D093	D16	B07	2917512	106.2	15.6	53.1	37.8	22.0	12.8	8.4	5.1	3.8	2.4
8	D071	N15	B08	2891543	105.7	15.7	52.4	40.9	28.9	19.5	12.7	6.8	4.3	4.5
9	D071	D17	B09	2891549	90.9	27.5	50.4	42.3	31.2	22.7	13.6	7.9	5.2	1.7
10	D014	N5	B10	927771	111.0	18.2	46.7	36.5	26.9	19.1	9.6	4.9	2.7	0.6
11	D054	L7	B11	710908	100.9	23.5	43.2	33.7	23.5	15.5	9.7	4.9	3.7	2.6
12	D090	D21	B12	799128	84.6	33.7	40.7	29.1	22.8	18.8	16.4	14.6	17.3	7.4
13	D019	A23	B13	6794179	103.7	24.0	39.8	31.6	24.9	18.1	12.2	9.4	4.3	0.9
14	D035	F14	B17	225394	121.2	10.9	38.0	26.9	18.6	9.8	5.8	3.6	1.2	1.4
15	D081	E9	B14	2913340	112.3	15.7	26.5	18.1	11.5	9.7	5.6	2.6	3.3	1.1
16	D004	F10	B15	2831046	125.0	10.6	25.2	16.8	12.6	5.2	5.4	2.1	0.1	-1.3
17	D011	L8	B16	2834881	117.0	13.9	22.9	14.6	10.1	3.5	1.2	0.3	-0.6	-0.5
