# Supplementary material for: Identification of novel small molecule Beclin 1 mimetics activating autophagy
Source: Oncotarget. 2017 May 18;8(31):51355–69. doi: 10.18632/oncotarget.17977 (PMC5584254; doi:10.18632/oncotarget.17977)
Supplement: Supplementary file 2 [file oncotarget-08-51355-s002.docx]

**Supplementary Table 2:** 41 hits from Microsource library, Prestwick library and CMLD library, with name, CID number, code, percentage of inhibition at 40 µM from primary screen, and dose-response reconfirmation data from secondary screen.

|  | **Plate ID** | **Coordinates** | **Name** | **Code** | **CID** | **Primary Screen** | | **Dose-response (% Inhibition)** | | | | | |
| --- | --- | --- | --- | --- | --- | --- | --- | --- | --- | --- | --- | --- | --- |
|  |  |  |  |  |  | mP | % Inhibition | 160 μM | 80 μM | 40 μM | 20 μM | 10 μM | 5 μM |
| 1 | D317 | K8 | RIBOFLAVIN | A31 | 493570 | -3.2 | 102.32 | 102.6 | 102.5 | 99.7 | 102.6 | 77.7 | 67.6 |
| 2 | D317 | H21 | SALICYLANILIDE | A39 | 6872 | -3.2 | 102.32 | 102.6 | 102.5 | 99.7 | 102.6 | 102.6 | 102.6 |
| 3 | D317 | N9 | TOPOTECAN HYDROCHLORIDE | A23 | 60699 | 43.8 | 68.69 | 102.6 | 101.7 | 70.9 | 64.7 | 54.8 | 39.8 |
| 4 | D321 | A4 | ACRIFLAVINIUM HYDROCHLORIDE | A26 | 6842 | -3.2 | 102.40 | 102.4 | 102.0 | 102.5 | 102.5 | 102.5 | 102.5 |
| 5 | D321 | D16 | PROFLAVINE HEMISULFATE | **A13** | 11111 | -3.2 | 102.40 | 102.4 | 102.4 | 102.5 | 101.3 | 65.8 | 55.9 |
| 6 | D322 | H11 | HARMALOL HYDROCHLORIDE | A28 | 5459187 | 56.2 | 60.00 | 86.2 | 73.0 | 56.8 | 41.6 | 31.2 | 19.2 |
| 7 | D323 | C11 | BRAZILEIN | A01 | 6453902 | 96.0 | 31.01 | 74.8 | 57.8 | 35.7 | 23.0 | 16.9 | 8.0 |
| 8 | D318 | M5 | DIPYRIDAMOLE | A22 | 3108 | 72.7 | 47.77 | 72.4 | 62.9 | 46.7 | 35.1 | 21.0 | 10.1 |
| 9 | D318 | H13 | QUINACRINE HYDROCHLORIDE | **A21** | 6239 | 72.2 | 48.17 | 66.3 | 58.3 | 47.9 | 38.6 | 26.7 | 14.5 |
| 10 | D321 | C3 | ETHACRIDINE LACTATE | **A15** | 15789 | 83.5 | 37.87 | 60.9 | 47.7 | 20.0 | 14.9 | 6.2 | -0.5 |
| 11 | D318 | G9 | DAUNORUBICIN | A36 | 30323 | 75.5 | 45.75 | 56.0 | 51.9 | 44.4 | 41.0 | 33.4 | 22.8 |
| 12 | D325 | G18 | DOXORUBICIN HYDROCHLORIDE | A25 | 443939 | 75.9 | 43.98 | 53.2 | 48.6 | 41.7 | 36.5 | 29.1 | 18.9 |
| 13 | D300 | I19 | KU0104511 | A37 | 17756814 | 90.6 | 30.11 | 47.6 | 38.4 | 28.7 | 20.1 | 13.9 | 7.7 |
| 14 | D319 | P6 | EPIRUBICIN HYDROCHLORIDE | A24 | 65348 | 136.0 | 22.76 | 40.9 | 34.4 | 23.1 | 16.2 | 11.3 | 6.8 |
| 15 | D321 | M11 | HOMIDIUM BROMIDE | A12 | 14710 | 113.8 | 15.31 | 39.2 | 26.1 | 12.0 | 7.2 | 5.5 | 3.5 |
| 16 | D319 | A23 | CHLORANIL | A27 | 8371 | 140.9 | 27.76 | 35.9 | 28.5 | 23.4 | 22.6 | 19.4 | -3.0 |
| 17 | D325 | M11 | MITOXANTRONE HYDROCHLORIDE | A11 | 51082 | 99.8 | 26.31 | 32.2 | 32.9 | 19.5 | 11.2 | 4.3 | -2.2 |
| 18 | D317 | E9 | MEFEXAMIDE HYDROCHLORIDE | A32 | 3083802 | 119.7 | 14.40 | 32.1 | 23.1 | 11.2 | 4.3 | 2.6 | -1.5 |
| 19 | D317 | A11 | SANGUINARINE SULFATE | A05 | 13394149 | 130.6 | 6.61 | 31.1 | 20.6 | 8.4 | 2.0 | -0.2 | -1.4 |
| 20 | D320 | J19 | ERGOTAMINE TARTRATE | A18 | 5352539 | 122.3 | 11.03 | 25.6 | 15.6 | 5.8 | 3.3 | 0.3 | -0.2 |
| 21 | D321 | C9 | SODIUM TETRADECYL SULFATE | A06 | 23665772 | 121.5 | 9.56 | 24.4 | 15.4 | 2.6 | -1.0 | -3.8 | -7.3 |
| 22 | D318 | E10 | GENTIAN VIOLET | A08 | 11057 | 123.0 | 11.68 | 22.7 | 21.2 | 6.6 | 5.8 | 3.7 | 1.9 |
| 23 | D322 | M22 | GARCINOLIC ACID | A02 | 6710618 | 124.0 | 11.76 | 22.0 | 18.1 | 7.5 | 3.6 | 3.3 | 2.5 |
| 24 | D322 | I20 | AGARIC ACID | A19 | 12629 | 118.2 | 15.85 | 20.9 | 20.3 | 12.7 | 9.1 | 6.3 | 3.8 |
| 25 | D320 | G5 | SENNOSIDE A | A34 | 73111 | 119.9 | 12.80 | 20.9 | 22.5 | 12.0 | 6.3 | 4.7 | 0.5 |
| 26 | D322 | G15 | SENNOSIDE B | A35 | 91440 | 120.4 | 14.32 | 19.9 | 21.1 | 13.6 | 5.8 | 4.1 | 2.7 |
| 27 | D317 | L5 | DANTHRON | A03 | 2950 | 126.4 | 9.62 | 19.7 | 8.7 | 4.9 | 4.8 | 1.4 | -1.2 |
| 28 | D326 | O6 | PROPIDIUM IODIDE | A40 | 104981 | 123.6 | 7.08 | 18.3 | 12.1 | 4.4 | 1.2 | -0.2 | 0.5 |
| 29 | D322 | C21 | GOSSYPOL | A38 | 3503 | 125.4 | 10.75 | 18.3 | 14.7 | 8.4 | 5.4 | 4.8 | 2.7 |
| 30 | D301 | F15 | KU0105065 | A33 | 16746258 | 117.5 | 5.69 | 15.8 | 8.3 | 6.3 | 3.2 | 3.6 | 2.4 |
| 31 | D303 | O15 | KU0104269 | A41 | 17756824 | 117.5 | 6.35 | 14.2 | 9.6 | 4.2 | 4.0 | 3.9 | 1.8 |
| 32 | D326 | M17 | BENZETHONIUM CHLORIDE | A07 | 8478 | 120.2 | 9.66 | 13.7 | 6.4 | 3.4 | 2.1 | -0.1 | -3.1 |
| 33 | D323 | C16 | LOBARIC ACID | A04 | 73157 | 129.2 | 7.13 | 13.7 | 10.2 | 3.2 | 2.0 | 2.3 | 1.4 |
| 34 | D310 | E17 | KU0157865 | A29 | 51360531 | 118.2 | 11.57 | 13.3 | 12.2 | 4.9 | 5.3 | 2.8 | 0.1 |
| 35 | D322 | J11 | CHOLIC ACID, METHYL ESTER | A09 | 10960835 | 127.0 | 9.59 | 13.2 | 9.3 | 4.2 | 1.7 | 1.5 | 1.6 |
| 36 | D320 | E17 | BENZBROMARONE | A20 | 2333 | 125.0 | 9.05 | 11.3 | 6.1 | 1.1 | 1.0 | 0.6 | 0.2 |
| 37 | D310 | M14 | KU0157983 | A30 | 51360535 | 122.8 | 8.15 | 11.2 | 8.8 | 5.3 | 4.4 | 1.6 | 0.7 |
| 38 | D327 | N10 | TELMISARTAN | A16 | 65999 | 125.4 | 5.20 | 11.1 | 6.3 | 1.6 | -0.1 | -0.6 | -1.1 |
| 39 | D317 | J21 | NONOXYNOL-9 | A17 | 72385 | 127.2 | 9.04 | 11.1 | 12.9 | 5.3 | 5.9 | 6.2 | -0.3 |
| 40 | D317 | M11 | FENBENDAZOLE | A10 | 3334 | 128.0 | 8.47 | 8.8 | -0.4 | -0.1 | 0.2 | 2.3 | -3.4 |
| 41 | D323 | L12 | AURIN TRICARBOXYLIC ACID | A14 | 2259 | 129.7 | 6.76 | 8.6 | 8.1 | 4.8 | 3.8 | 2.0 | 0.7 |
